# Trend in basic oral treatment needs in relation to taxation of sweets, ice cream, and sugar-sweetened beverages in Finland: a registry-based study

**DOI:** 10.2340/aos.v83.40335

**Published:** 2024-04-16

**Authors:** Jesse Jokirinta, Jari Päkkilä, Evangelos Mourelatos, Sakari Sipola, Marja-Liisa Laitala, Saujanya Karki

**Affiliations:** a Research Unit of Population Health, University of Oulu, Oulu, Finland; b Research Unit of Mathematical Sciences, University of Oulu, Oulu, Finland; c Oulu Business School, University of Oulu, Oulu, Finland; d Medical Research Center Oulu and Oulu University Hospital, Oulu, Finland

**Keywords:** sugar, sugar-sweetened beverages, taxation, costs, treatment need

## Abstract

**Objective:**

The aims were to explore the trend in basic oral treatment needs and total operating cost of public dental services (PDS) in relation to total excise tax revenue generated from sugary products during 2011–2020 and to evaluate the impact of tax policy in excise tax revenue of sugary products and average sugar consumption.

**Methods:**

The study comprised longitudinal data retrieved from Finnish registries during the years 2011–2020. Basic oral treatment needs, and total operating cost of PDS, total excise tax revenue generated from sugary products and average sugar consumed (kg per capita) during the years 2011–2020 were obtained. Simplified panel analyses and sensitivity analyses were used to evaluate the effects of explanatory variables on outcomes.

**Results:**

An approximate one EUR 1,000,000 increase in total excise tax revenue generated from sugary products corresponds to a 0.4% increase in total operating cost of PDS. There was a significant positive trend in total operating cost of PDS in Finland over the study period. Similarly, an approximate one EUR 1,000,000 rise in total excise tax revenue corresponds to a 0.2% increase in basic oral treatment needs. Additionally, there was a statistically significant difference in the average excise tax revenue for sugary products between the periods before and after 2017.

**Conclusion:**

No change in average sugar consumption was observed despite implementing the new sugar policy. Therefore, it may be worthwhile to reconsider the excise tax on sweets and ice cream as it will significantly increase the total national revenues.

## Introduction

Added sugars include sugars added to food or beverages while food processing [1]. Added sugars are commonly found in table sugar, sugar-sweetened beverage (SSB), candies, and confectionaries. Frequent and excessive intake of added sugar is one of the common risk factors for non-communicable diseases (NCD) such as obesity, diabetes, cardiovascular diseases as well as dental caries [1]. Studies have reported a strong correlation between the amount of sugar consumption [2] and SSB and dental caries increment [3]. Likewise, increased sugar intake is also associated with periodontal diseases [4]. In Finland, mean enamel caries is high among adults [5], and mean dental caries seem to be plateaued during 2000 and 2011 [6]. Moreover, the polarization of dental caries still exists among Finns [5, 7], and periodontitis is still high, especially among young Finnish men [6]. Noteworthily, the coexistence of dental caries and periodontal diseases in Finnish adults was reported earlier [8].

In Finland, oral health care services are provided by the public dental service (PDS) and private sector. Children and adolescents (under 18 years old) have been entitled to receive free of charge care (regular examinations, preventive care and treatment including necessary orthodontic treatments) in the PDS since 1950s. However, since 2002, all the residents have been granted access to the PDS or to the Social Insurance Institution of Finland (KELA) led partial reimbursed private oral health care services. There is a fixed fee-for-services only in the PDS but not in the private sector. Additionally, if required, primary oral health care services are sometimes purchased by public healthcare providers from private sectors [9, 10]. The Finnish Student Health Services organises primary health care, including oral health care, for students attending higher education institutes [11].

The Finnish law on excise duty on sugar came into the force for the first time in 1940. During that period, raw or refined sugar products made from sugar beet were taxed. Later, in 2011, the Finnish law on sweets, ice cream, and SSBs (sugary products) came into force. The levied tax on SSB is constantly changing (e.g. 11 cnt/L in 2011 to 27 cnt/L in 2019); however, after 2017, sweets and ice cream (95 cnt/kg in 2011) no longer had excise tax [12]. The amount of tax depends upon the sugar content on products. Recently, the tax on sugar-free drinks decreased from 13 to 9 cnt/L. However, the tax on drinks containing added sugar or only natural sugar ranged from 16 to 48 cnt/L [13].

The average sugar consumption among Finns in 2020 was 32.2 kilograms per capita [14], and the domestic sales of soft drinks were 278.4 million litres in the year 2020 [15]. A study reported that the taxation of sugar and SSB had played a major role in reducing the consumption of SSBs in Finland [16]. Previously, Bíró [17] concluded that with increased sugar taxation, the consumption of processed food (with added sugar) decreased by 3.4%. Similarly, in scenario analyses done in Germany [18], Australia [19], and the Netherlands [20] significant reduction of dental caries was predicted by implementing a 20% sales tax on SSB. However, no studies were conducted earlier to evaluate the relation between the taxation of sugary products and oral treatment needs as well as operating cost of PDS in Finland.

Therefore, the aim of this study is to explore the trend in basic oral treatment needs and total operating cost of PDSs in relation to total excise tax revenue generated from sugary products during 2011–2020. Another aim was to evaluate the impact of tax policy (2017) in excise tax revenue of sugary products and average sugar consumption. The study hypotheses were that there is a negative trend between taxation and oral treatment needs (i.e. increase in excise tax revenue of sugary products will decrease basic oral treatment needs and total operating cost of PDSs) and sugar policy (2017) has an impact on excise tax revenue and average sugar consumption.

## Materials and methods

This was a nationwide register-based study in Finland that comprised longitudinal data retrieved from registries during the period of 2011–2020.

### Study variables

This study included the basic oral treatment needs and total operating cost of PDSs during the years 2011–2020 that were obtained from the Finnish Institute for Health and Welfare. The need for basic oral treatment is defined as the proportion of patients in need for dental or periodontal care (i.e. presence of one or more decayed teeth and/or community periodontal index [CPI] score 3 or more). Likewise, the total operating cost of PDSs is defined as direct cost required to organise oral health care services in Finland (EUR 1,000,000 per year) [21]. Similarly, the excise tax revenue generated from sweets, ice cream, and SSBs (EUR 1,000,000 per year) during the years 2011–2020 was obtained from Statistics Finland [22], and average sugar consumed (kilogram per capita) during the years 2011–2020 was obtained from the Natural Resources Institute Finland [14].

### Ethical consideration

Ethical clearance was not considered necessary because the study was done using secondary data from open registries.

### Statistical analyses

Data on the basic oral treatment needs, total operating costs of PDSs, total excise tax revenue generated from sugary products, and average sugar consumption during the years 2011–2020 were combined into Microsoft Excel®. All statistical analyses were done using the R program (R Core Team, 2021) and Stata Statistical Software 18 (StataCorp LLC, 2023).

To explore the effects of total excise tax revenue generated from sugary products (EUR 1,000,000 per year) and the average sugar consumption (kg per capita) on total operating cost of PDSs (in EUR 1,000,000 per year) and the basic oral treatment needs (percentage), simplified panel analyses were used, by taking into consideration of the temporal trend (2011–2020) within the following specification:


$${\text{Y}}_{\text{i}}=\text{α}+\text{β}{\text{X}}_{\text{i}}+\text{γ}{\text{Z}}_{\text{i}}+\text{δ}{\text{T}}_{\text{i}}+{\text{e}}_{\text{i}}$$ (1)

where Y_i_ is two dependent variables in each case (e.g. logarithmic values of total operating cost of PDSs and the percentage of basic oral treatment needs) in Finland in year i. X is total excise tax revenue generated from sugary products (EUR 1,000,000 per year), Z is the average sugar consumption and T is a vector including the temporal trend in years (2011–2020). Lastly, e_i_ is the disturbance term.

When analyzing the total operating cost of PDSs, the values were transformed into a logarithmic scale to facilitate more meaningful interpretations when using the Ordinary Least Squares (OLS) regression model. In the context of estimating basic oral treatment needs, a fractional probit model was employed, given that the outcome is represented as a percentage. Fractional probit models are designed to predict the conditional mean of the dependent variable ’Y’ based on the covariates ’X.’ Since ’Y’ falls within the range of 0 to 1, it is advisable to report the marginal effects for a more informative analysis.

Additionally, to understand the impact of a new sugar tax policy (2017) on the independent variables, sensitivity analyses were performed. To gain a deeper insight into these changes, a methodology to track the percentage change of these variables over time was devised. This involved subtracting the earlier index value from the later one, then dividing this difference by the earlier index value. The final step is to multiply the result by 100. To confirm the trends, difference in mean independent *t*-tests with a 5% confidence level were performed.

## Results

**Figure 1:**
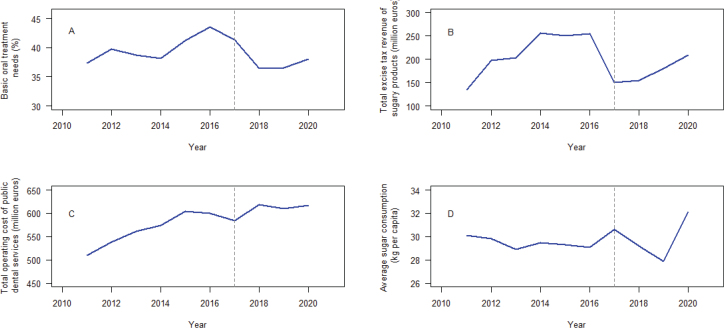
Linear graphs for basic oral treatment needs, total operating cost of public dental services, total excise tax revenue generated from sugary products and average sugar consumed during the years 2011-2020

Figure 1 depicts the linear graphs plotted during the years 2011–2020 for basic oral treatment needs, total operating cost of PDSs, total excise tax revenue generated from sugary products and average sugar consumed.

Table 1 displays the estimates for two key factors: the total operating cost of PDSs (expressed in logarithmic values in column 1) and the basic oral treatment needs (as a percentage in column 2). Notably, there was a statistically significant positive association between the total excise tax revenue generated from sugary products and the total operating cost of PDSs. Specifically, an approximate one EUR 1,000,000 increase in total excise tax revenue corresponds to a 0.4% increase in the total operating cost of PDSs. When examining temporal trends, an annual increase of 1.9% in the total operating cost of PDSs was observed. However, there was no effect of average sugar consumption on the total operating cost of PDSs.

**Table 1:** Association between basic oral treatment needs (proportion) and total operating cost of public dental services (in million euros) in relation to total excise tax revenue of sugary products (in million euros) during 2011 – 2020 in Finland.

Explanatory variables	Total operating cost of public dental services (in million euros)	Basic oral treatment needs (in proportion)
	[1]	[2]
Total excise tax revenue	0.004** (0.001)	0.002* (0.001)
Average sugar consumption	−0.090 (0.060)	0.030 (0.005)
Years	0.019*** (0.002)	-0.010 (0.008)
*R*-squared	0.929	
*F*-Stat	26.22	
Pseudo *R*-squared		0.401
Wald chi^2^		14.12

Source: Data drawn from [14, 21, 22].

Notes: ***N*** = 10. Column 2 reports marginal effects. Robust Standard errors are in parentheses.

* ***p*** < 0.1;

** ***p*** < 0.05

*** ***p*** < 0.01.

Concerning the impact on basic oral treatment needs, a statistically significant positive association between total excise tax revenue generated from sugary products was observed, albeit at a 10% significance level. In particular, an approximate one EUR 1,000,000 rise in total excise tax revenue generated from sugary products corresponds to a 0.2% increase in yearly basic oral treatment needs. No additional effects related to average sugar consumption or temporal trends are observed in relation to basic oral treatment needs (Table 1).

**Figure 2:**
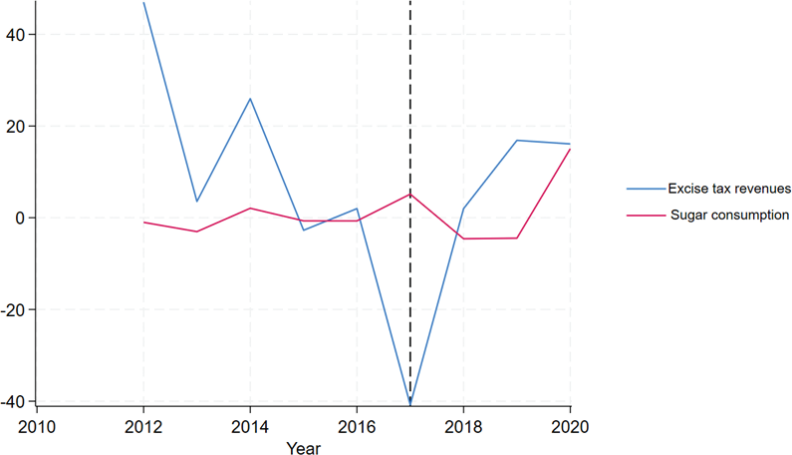
Percentage change of total excise tax revenue of sugary products (in million euros) and average sugar consumption (kg per capita) during the study period.

Figure 2 illustrates the change in total excise tax revenue generated from sugary products and average sugar consumption during the study period. It is worth noting that sugar consumption remained relatively stable, although were no longer subject to excise tax as of 2017, while the total excise tax revenue generated from sugary products experienced a significant percentage decrease.

Table 2 confirms these trends, by comparing the mean of these variables before and after 2017. By using paired *t*-tests, we have shown that, there was a statistically significant difference in the average excise tax revenue generated from sugary products, measured in EUR 1,000,000, between the periods before and after 2017. This difference indicates a decrease over time.

**Table 2:** Mean change of total excise tax revenue generated from sweets, ice cream, and sugar-sweetened beverages (in million euros) and average sugar consumption (kg per capita) before and after 2017.

Variables	Paired variables			
	Before 2017	After 2017	Difference [2]–[1]	*t*-test |t|
	[1]	[2]	[3]	[4]
Total excise tax revenue	216.16	173.50	−42.66	1.60**
Average sugar consumption	29.45	29.95	−0.50	0.67

Source: Data drawn from [14, 22]

Note:

** ***p*** < 0.05

## Discussion

This study aimed to explore the trend in basic oral treatment needs and total operating cost of PDSs in relation to total excise tax revenue generated from sugary products during 2011–2020. Another aim was to evaluate the impact of tax policy (2017) in excise tax revenue of sugary products and average sugar consumption. This study revealed a positive association between the total excise tax revenue generated from sugary products and the total operating cost of PDSs as well as basic oral treatment needs. However, there were no effects of average sugar consumption on both total operating cost of PDSs and basic oral treatment needs. During 2011–2020, sugar consumption remained relatively stable in Finland, although sweets and ice cream were no longer subject to excise tax as of 2017, while the total excise tax revenue generated from sugary products experienced a significant percentage decrease. However, an annual increase of 1.9% in the total operating cost of PDSs was observed during the study period.

One of the limitations of this study is the reliability of the registries-based clinical data. According to Finnish legislation, public and private dental services are obliged to update their patient records in the national registries [23], and good reliability among dentists in public health care registering oral health data was previously reported [24]. Another limitation would be estimating average sugar consumption without considering the population variance (such as age group). Therefore, a cautious interpretation of the results from the current study is required. However, the result of this study can be generalised in Finland as all the variables were obtained from the national registries of Finland.

In the present study, an association between total excise tax revenue generated from sugary products and total operating cost of PDS was found. In Finland, local authorities (municipalities until 2022 and well-being Service Counties from 2023) are obliged to organise social and healthcare services to their inhabitants [25] and public health care financing is provided by state and municipal taxes, national health insurance contribution, and out-of-pocket payments. It can be presumed that the national revenues in Finland have increased during the study period and in turn increased the spending in public health care including dental care *per se*. A previous study also concluded that the increase in tax revenue supports the government on their health expenditure [26]. For example, the total government revenue from production and imports in Finland has increased from 5.3 billion euros in 2011 to 10.0 billion euros in 2020 [22] and health expenditure per person has also doubled in the past two decades (US$ 1,900 in 2000 and US$ 4,897 in 2020) [27]. Additionally, a significant positive trend in the total operating costs of PDS was observed in the present study. As expected, to mitigate the oral health care demand as per population growth and need due to dental care reform in 2001–2002 may have increased the operating cost in Finland. For instance, the number of adults visiting the PDS in the year 2013 increased by 81.5% compared to the year 2001 [28]. This was also supported by a recent study [29].

The positive association between the total excise tax revenue generated from sugary products and basic oral treatment needs was revealed in the present study. In contrast, previous studies have predicted reduction of dental caries by implementing a 20% sales tax on SSB [18–20]. One of the reasons for small increment in proportion of basic oral treatment needs over the time may be due to an increase in average sugar consumption, despite being statistically significant. Another could be due to increase in the number of adults age group visiting PDSs in Finland [28, 29]. For example, health surveys showed an increase in proportion of those requiring periodontal treatment, especially among 30–44-year-olds [6]. However, another study concluded that there was a decreasing trend in basic treatment needs among adults [28]. To rule out the relationship between excise tax revenue and oral treatment needs, further studies that incorporate other health hazard products (such as tobacco) must be conducted. Additionally, other adverse health effects must also be studied.

Nevertheless, the long-term and excessive consumption of SSB is associated with morbidity and mortality [30]. A recent report also showed that dietary factors, including high SSB consumption, were responsible for almost two-fifths of all deaths in 2017 in Finland [31]. According to the Finnish law (2013), an excise tax (13 cents/L) is also levied on natural mineral bottled waters without free sugar and abolished from sweets [13]. Therefore, the adverse health effects of these products should be investigated, and policy should be reviewed, if necessary. Another measure would be introducing warning labels on sugar and SSB to reduce the intake [32].

In conclusion, despite limitations, an increasing trend in total operating cost of PDS was found in the present study that is in line with government policies. Additionally, no change in average sugar consumption was observed despite implementing the new sugar policy (2017). Therefore, it may be worthwhile to reconsider the excise tax on sweets and ice cream as it will significantly increase the total national revenues. These revenues could be further invested in promoting oral and general health in Finland.

## Declaration of interest

The authors declare that they have no competing interests to declare.

## References

[1] World Health Organization. Guideline: sugars intake for adults and children. 2015. [cited 2022 Dec 22]. Available from: https://apps.who.int/iris/handle/10665/149782 25905159

[2] Bernabé E, Vehkalahti MM, Sheiham A, et al. The shape of the dose-response relationship between sugars and caries in adults. J Dent Res. 2016;95(2):167–72. 10.1177/0022034515616572 26553884

[3] Bernabé E, Vehkalahti MM, Sheiham A, et al. Sugar-sweetened beverages and dental caries in adults: a 4-year prospective study. J Dent. 2014;42(8):952–8. 10.1016/j.jdent.2014.04.011 24813370

[4] Kusama T, Nakazawa N, Takeuchi K, et al. Free sugar intake and periodontal diseases: a systematic review. Nutrients. 2022;14(21):4444. 10.3390/nu14214444 36364708 PMC9656760

[5] Laajala A, Pesonen P, Anttonen V, et al. Association of enamel caries lesions with oral hygiene and DMFT among adults. Caries Res. 2019;53(4):475–81. 10.1159/000497358 30917373

[6] Suominen AL, Varsio S, Helminen S, et al. Dental and periodontal health in Finnish adults in 2000 and 2011. Acta Odontol Scand. 2018;76(5):305–13. 10.1080/00016357.2018.1451653 29546776

[7] Tanner T, Kämppi A, Päkkilä J, et al. Prevalence and polarization of dental caries among young, healthy adults: cross-sectional epidemiological study. Acta Odontol Scand. 2013;71(6):1436–42. 10.3109/00016357.2013.767932 23627898

[8] Mattila PT, Niskanen MC, Vehkalahti MM, et al. Prevalence and simultaneous occurrence of periodontitis and dental caries. J Clin Periodontol. 2010;37(11):962–7. 10.1111/j.1600-051X.2010.01620.x 20958340

[9] Niiranen T, Widström E, Niskanen T. Oral Health Care Reform in Finland – aiming to reduce inequity in care provision. BMC Oral Health. 2020 Apr 21;20(1):121. 10.1186/1472-6831-8-3 18226197 PMC2268684

[10] Suominen AL, Helminen S, Lahti S, et al. Use of oral health care services in Finnish adults – results from the cross-sectional Health 2000 and 2011 Surveys. BMC Oral Health. 2017;17(1):78. 10.1186/s12903-017-0364-7 28438160 PMC5402661

[11] Finlex. Law on student health care for university students 2019. [cited 2023 Oct 24]. Available from: https://www.finlex.fi/fi/laki/alkup/2019/20190695

[12] Finlex. Law on soft drink tax 2010. [cited 2021 Feb 10]. Available from: https://www.finlex.fi/fi/laki/ajantasa/2010/20101127

[13] Finlex. Law on soft drink tax 2023. [cited 2023 Oct 23]. Available from: https://www.finlex.fi/fi/laki/alkup/2023/20230651

[14] Natural Resources Institute Finland. Balance sheet for food commodities. [cited 2021 Oct 9]. Available from: http://statdb.luke.fi/PXWeb/pxweb/fi/LUKE/LUKE__02%20Maatalous__08%20Muut__02%20Ravintotase/01_Elintarvikkeiden_kulutus.px/table/tableViewLayout2/?rxid=dc711a9e-de6d-454b-82c2-74ff79a3a5e0

[15] Brewery and Soft Drink Industry Association. Domestic sales statistics. [cited 2022 Dec 16]. Available from: https://panimoliitto.fi/tilastot/myyntitilastot/

[16] Heinonen M. The Finnish excise tax on sugar-sweetened beverages and its effect on prices and demand. 2018. Master thesis. University of Jyväskylä.

[17] Bíró A. Did the junk food tax make the Hungarians eat healthier? Food Policy. 2015;54:107–15. 10.1016/j.foodpol.2015.05.00

[18] Schwendicke F, Thomson WM, Broadbent JM, et al. Effects of taxing sugar-sweetened beverages on caries and treatment costs. J Dent Res. 2016;95(12):1327–32. 27671690 10.1177/0022034516660278

[19] Sowa PM, Keller E, Stormon N, et al. The impact of a sugar-sweetened beverages tax on oral health and costs of dental care in Australia. Eur J Public Health. 2019;29(1):173–7. 10.1093/eurpub/cky087 29796599

[20] Jevdjevic M, Trescher AL, Rovers M, et al. The caries-related cost and effects of a tax on sugar-sweetened beverages. Public Health. 2019;169:125–32. 10.1016/j.puhe.2019.02.010 30884363

[21] Finnish Institute for Health and Welfare. Statistical information on welfare and health in Finland. [cited 2021 Feb 10]. Available from: https://sotkanet.fi/sotkanet/en/index

[22] Statistics Finland. StatFin Databases. [cited 2022 Jan 1]. Available from: https://www.stat.fi/tup/statfin/index_en.html

[23] Finlex. Decree of Ministry of Social Affairs and Health from patient documents. [cited 2022 Dec 22]. Available from: https://www.finlex.fi/fi/laki/alkup/2022/20220094

[24] Hausen H, Kärkkäinen S, Seppä L. Caries data collected from public health records compared with data based on examinations by trained examiners. Caries Res. 2001;35(5):360–5. 10.1159/000047475 11641572

[25] Finlex. Health Care Act. [cited 2023 Oct 24]. Available from: https://www.finlex.fi/en/laki/kaannokset/2010/en20101326

[26] Reeves A, Gourtsoyannis Y, Basu S, McCoy D, McKee M, Stuckler D. Financing universal health coverage – effects of alternative tax structures on public health systems: cross-national modelling in 89 low-income and middle-income countries. Lancet. 2015;386(9990):274–80. 10.1016/S0140-6736(15)60574-8 25982041 PMC4513966

[27] Keskimaki I, Tynkkynen LK, Reissell E, et al. Finland: health system review. Health Syst Transit. 2019;21(2):1–166. 31596240

[28] Linden J, Widström E, Sinkkonen J. Adults’ dental treatment in 2001-2013 in Finnish public dental service. BMC Oral Health. 2008 Jan 28;8:3. 10.1186/s12903-020-01091-w 32316958 PMC7171728

[29] Raittio E, Suominen AL. Effects of universal oral healthcare coverage in an adult population: a long-term nationwide natural experiment. Community Dent Oral Epidemiol. 2023;51(5):908–17. 10.1111/cdoe.12785 36036466

[30] Malik VS, Li Y, Pan A, et al. Long-term consumption of sugar-sweetened and artificially sweetened beverages and risk of mortality in US adults. Circulation. 2019;139(18):2113–25. 10.1161/CIRCULATIONAHA.118.037401 30882235 PMC6488380

[31] European Observatory on Health Systems and Policies. Finland: country health profile 2021. State of Health in the EU. Paris: OECD Publishing; 2021 [cited 2022 Dec 22]. Available from: 10.1787/2e74e317-en

[32] Leung C W, Wolfson JA, Hsu R, et al. Warning labels reduce sugar-sweetened beverage intake among college students. J Nutr. 2021;151(1):179–185. 10.1093/jn/nxaa305 33245125 PMC7779215

